# An Interactive Voice Response System to Increase Physical Activity and Prevent Cancer in the Rural Alabama Black Belt: Design and Usability Study

**DOI:** 10.2196/29494

**Published:** 2022-01-04

**Authors:** Mohanraj Thirumalai, Nashira Brown, Soumya Niranjan, Sh'Nese Townsend, Mary Anne Powell, Whitney Neal, Erica Schleicher, Venkatadri Raparla, Robert Oster, Wendy Demark-Wahnefried, Dori Pekmezi

**Affiliations:** 1 Department of Health Services Administration School of Health Professions University of Birmingham at Alabama Birmingham, AL United States; 2 Department of Nutrition Sciences School of Health Professions University of Birmingham at Alabama Birmingham, AL United States; 3 Department of Health Behavior School of Public Health University of Alabama at Birmingham Birmingham, AL United States; 4 University of Alabama at Birmingham/Lakeshore Research Collaborative School of Health Professions University of Alabama at Birmingham Birmingham, AL United States; 5 Division of Preventive Medicine Heersink School of Medicine University of Alabama at Birmingham Birmingham, AL United States

**Keywords:** interactive voice response systems, usability, exercise, physical activity, rural health, telehealth

## Abstract

**Background:**

Increased physical activity (PA) levels are associated with reduced risk and improved survival for several cancers; however, most Americans engage in less than the recommended levels of PA. Using interactive voice response (IVR) systems to provide personalized health education and counseling may represent a high-reach, low-cost strategy for addressing physical inactivity and cancer disparities in disproportionately burdened rural regions. However, there has been a paucity of research conducted in this area to date.

**Objective:**

The aim of this study is to design, develop, and test the usability of an IVR system aimed at increasing PA levels in the rural Alabama Black Belt.

**Methods:**

A pilot version of the IVR system was used to assess initial feasibility and acceptability. Detailed exit interviews were conducted to elicit participant feedback, which helped inform the development of a substantially upgraded in-house IVR system. This refined IVR system was then subjected to a sequential explanatory mixed methods evaluation. Participating rural county coordinators and research staff (N=10) tested the usability of the IVR system features for 2 weeks and then completed the System Usability Scale and qualitative semistructured interviews.

**Results:**

The study sample comprised mostly African American people, women, rural county coordinators, and research staff (N=10). Participants rated the IVR system with a mean score of 81 (SD 5) on the System Usability Scale, implying *excellent* usability. In total, 5 overarching themes emerged from the qualitative interviews: likes or dislikes of the intervention, barriers to or facilitators of PA, technical difficulties, quality of calls, and suggestions for intervention improvement. Message framing on step feedback, call completion incentives, and incremental goal-setting challenges were areas identified for improvement. The positive areas highlighted in the interviews included the personalized call schedules, flexibility to call in or receive a call, ability to make up for missed calls, narration, and PA tips.

**Conclusions:**

The usability testing and feedback received from the rural county coordinators and research staff helped inform a final round of refinement to the IVR system before use in a large randomized controlled trial. This study stresses the importance of usability testing of all digital health interventions and the benefits it can offer to the intervention.

## Introduction

### Background

Automated telephone-based intervention strategies may be key to overcoming the numerous barriers to physical activity (PA) promotion and cancer control in the Alabama Black Belt, a rural region named for its rich soil but whose population is at increased risk for sedentary lifestyles and related cancer disparities [1]. Low literacy, poverty, lack of transportation, cultural preferences, and distance from PA facilities often impede access to PA information and resources in this region [2]. Interactive voice response (IVR) systems allow users to interact by pressing keys on the telephone keypad and can be effective in targeting behavior change [3]. The recent National Health Interview Survey estimates that only 0.7% of the population in the United States is phoneless [4], thereby demonstrating the potential for a wider reach of IVR interventions. Moreover, IVRs do not require clinic visits, high literacy, or access to costly technology [5,6].

In response, we have developed an IVR-delivered PA intervention that is currently being tested in a large randomized controlled trial (RCT) in 6 rural Black Belt counties of Alabama. This paper describes the process that led us to the design of the IVR system and the results of the usability testing that was conducted before the commencement of the RCT.

As with any intervention, particularly digital health interventions, examination of the usability of the developed intervention before the actual deployment of the intervention is vital [7]. With IVR systems featuring only voice-based output and keypad-based input, a seamless user experience can indeed be tricky [8-10]. IVR-based intervention systems can pose more challenges than simple IVR data collection systems, as IVR-based intervention systems need to focus on achieving minimal information navigation time, while featuring maximal information relevance and capacity [8].

### Objectives

Our proposed study aims to target rural Black Belt counties of Alabama that are marked by low literacy and education levels [1,2]. Although there is a body of work focused on the usability of IVR systems [8-10], there is limited research on the usability of IVR systems for rural settings and underserved populations. This limited body of literature has used surveys and interviews to evaluate the usability of IVR systems. This study seeks to fill this gap in the literature by reporting our development methodology, system features, and explanatory sequential mixed methods design to assess the usability of the IVR system. Our hypotheses are that most participants in this usability study will rate the usability of the IVR system favorably and provide useful suggestions for further improvements during interviews.

## Methods

### Parent Study Overview

The parent study (R01CA233550) is an ongoing RCT (N=240) comparing a Deep South IVR-Supported Active Lifestyle (DIAL) intervention with a waitlist control among underactive adults residing in 6 rural Alabama counties [11]. On the basis of the social cognitive theory (SCT) [12], this study extends an IVR-supported PA intervention that targets key SCT constructs (self-regulation, self-efficacy, enjoyment, outcome expectations, and social support) through IVR counseling calls. The participants are provided pedometers (Accusplit AX2790MV) and Fitbit activity monitors (model: Inspire) to record daily steps and receive progress feedback via the IVR PA-tracking and goal-setting calls.

The number of calls in a week tapers as participants progress through the intervention (from daily calls in months 0-3 to twice per week in months 4-6 and weekly in months 7-12), and the content of the calls vary based on specific days of the intervention.

### Iterative IVR System Design

#### Piloting a Beta Version of IVR

A previous pilot study (R03CA177538) tested a beta version of this IVR system with a convenience sample (N=63) [13-15]. Findings from this trial supported the feasibility and acceptability of the approach and helped further refine the technology and theory-driven intervention components in preparation for extension to rural populations. More specifically, the findings yielded the need for IVR-initiated calls as opposed to only participant-initiated calls, specific targeting of unchanged SCT constructs and incorporating multi-level strategies (incremental goal-setting and county coordinator support) for increased support, accountability, and sustainability [14].

The IVR system used in this pilot study was a commercial IVR system and posed several limitations. First, all voice clips were prerecorded by voice narrators and uploaded. Second, the system only worked by participants calling into the system and did not offer a way for the system to initiate calls. The commercial system also posed limitations in terms of dynamic tailored questions that used earlier responses to frame newer questions as the call progressed.

#### Upgrading and Refining the IVR System

In response to this pilot study feedback, we developed a completely homegrown IVR system for the parent RCT using more up-to-date technology. This new system was hosted on a Linux server, powered by an Apache web server, programmed using the Laravel framework (a hypertext preprocessor–based rapid development framework), data stored using a MySQL database, and connected to Twilio for telephony.

Although the system was being developed, we conducted focus groups with multiple stakeholder groups (rural county coordinators and research staff from the University of Alabama at Birmingham [UAB] O’Neal Comprehensive Cancer Center Community Outreach and Engagement Office). For the focus groups, we generated 3 sample voice clips of intervention messages using Amazon Polly, a text-to-speech engine, and presented the 3 sample voice clips to our stakeholders. Amazon Polly is capable of close to human-like voices, which resulted in stakeholders preferring Amazon Polly voices over prerecorded human voices. This choice of Amazon Polly voices also allows for the use of different tones and genders for the voices during the calls and avoids the extensive time and financial costs associated with rerecording message libraries with human narrators every time an edit is made to the content.

The focus group participants also provided feedback on incoming versus outgoing calls, the preferred procedure to handle missed calls, and other support strategies. More specifically, they felt that their community members and potential participants would appreciate the flexibility and convenience of bidirectional calls and the option to fill in missed call data at later dates. The need to be able to change phone numbers and allow incoming calls from new (unregistered) numbers was stressed. Support strategies, such as brief counseling sessions during in-person data collection and offering Fitbit devices were also suggested.

The system development was conducted in an agile fashion, with regular demonstrations to the rural country coordinators from the UAB O’Neal Comprehensive Cancer Center Community Outreach and Engagement Office. Their feedback regarding the speed of the voice clips, pauses between sentences, pauses in sentences, length of the phone call, reading level of the language used in the calls, and logical flow of the content resulted in numerous edits. The system included a participant call completion incentive mechanism that awarded the participants a minimum of US $0.25 for each call completed. However, the incentive amount became US $0.50 when the participant completed 7 preceding calls, with the incentive falling back to US $0.25 when a call was not completed.

The development phase concluded with the core project staff (DP, MT, ST, and VR) pilot-testing the revised system to identify and fix any problems. Some examples of the problems identified and fixed include system expecting responses within 5 seconds, incorrect feedback messages, and outgoing calls not being placed as scheduled. After this, a formal usability test was conducted as detailed in the following section. Finally, the system went through another round of iterative refinements based on the findings from the usability testing. The details of the resultant system are presented in the *Results* section.

### Usability Testing

#### Study Design

This study incorporated an explanatory sequential mixed methods design to assess the usability of an IVR phone counseling system that will be extended to physically inactive residents in 6 rural Alabama counties (Hale, Choctaw, Greene, Marengo, Dallas, and Sumter). Demographics were assessed at baseline. System usability and semistructured interviews were conducted at the 2-week follow-up.

#### Participants

The sample for usability testing comprised 10 rural county coordinators and research staff affiliated with the UAB O’Neal Comprehensive Cancer Center Community Outreach and Engagement Office who would later serve a critical role in recruitment, assessment, and intervention delivery for the RCT study but had yet to be exposed to the newly developed IVR system.

#### Procedures

Each participant completed a one-on-one orientation via Zoom with the DIAL program manager or principal investigator. During the session, the participants were given an overview of the usability study protocols and the IVR system, completed an initial IVR call with the research team, and asked questions.

Following orientation, the participants began wearing a study-provided pedometer or an approved personal activity monitor (ie, Fitbit or Apple Watch) and receiving daily IVR calls from DIAL for 2 weeks. The participants received all 3 types of IVR calls: PA-tracking, goal-setting, and counseling calls. For tracking calls, the participants answered PA questions (reported pedometer use, steps per day, and any moderate-intensity PA in the past 24 hours) and received PA tips and feedback. Tracking calls lasted approximately 1 minute per call. During the counseling calls, the participants answered PA questions and additional questions covering PA self-efficacy, enjoyment, outcome expectations, and social support. Moreover, they received tailored feedback on these psychosocial variables based on their individual responses to these questions. Counseling calls lasted approximately 10 minutes per call. Goal-setting calls allowed the participants to set their own step goal or increase their current step goal by 500 steps for the upcoming week. Goal-setting calls lasted approximately 5 minutes per call. In the 2-week period, the participants received 1 call per day, with a total of 1 goal-setting call, 1 counseling call, and 12 PA-tracking calls.

#### Quantitative Measures

##### Demographics

Participant demographics, including age, gender, educational attainment, race and ethnicity, household income, employment, marital status, and number of children living at home were assessed at baseline.

##### Survey Items

At follow-up, the participants completed the System Usability Survey (10 items) on the web via Qualtrics XM (Qualtrics) combined with 4 more project-specific items. All 14 items were aimed at assessing how the participants felt about the phone counseling system after using it for 2 weeks. The participants responded to the statement—*Please select the answer that best expresses how you feel about each statement after using the phone counseling system over the past 2 weeks*—for items such as *I think I would like to use this phone counseling system frequently*, *I thought the phone counseling system was easy to use*, *I felt very confident using the phone counseling system*, and *I needed to learn a lot of things before I could get going with this phone counseling system*. The 4 project-specific items were worded as *How likely are you to recommend this system to others?*, *Did you receive your calls at the scheduled time?*, *What gender was the voice on your calls?*, and *Did you use the study-provided pedometer to track your steps?*

#### Quantitative Analysis

All quantitative data collected during this study were descriptively analyzed. Microsoft Excel was used for all the quantitative analyses.

#### Qualitative Methodology

After 2 weeks of receiving calls and completing the quantitative survey, all 10 rural county coordinators and research staff participated in one-on-one, semistructured interviews conducted via Zoom regarding their experiences with the calls and how usability could be improved before implementing the IVR for the RCT. The semistructured interview guide was developed by coauthors (DP and SN) and included questions regarding motivation to exercise, likes and dislikes of the calls, specific call features that could motivate or demotivate individuals, technical aspects of the IVR call, and suggestions for improvement. To ensure consistency, all interviews were conducted in July 2020 by 1 member of the study team (SN) with expertise and experience in qualitative interviewing. SN is not involved in any aspect of the broader RCT or technology design and development and was engaged to serve as a neutral evaluator for the purpose of this usability evaluation.

#### Qualitative Analysis

All interviews were audio-recorded and transcribed verbatim by a professional transcription service. Thematic analysis [16] was conducted using NVivo 13 (QSR International) [17]. Investigator triangulation methodology was conducted [18] by a 2-member analysis team (DP and SN) with experience in qualitative methodology in social science disciplines (clinical psychology and medical sociology) who independently reviewed transcripts through line-by-line coding. After the initial categories and themes were generated in a cyclical, iterative process, the full research team refined the existing categories, themes, and subthemes. Discrepancies, although infrequent, were addressed with the research team.

## Results

### Participant Characteristics

Participant characteristics are shown in Table 1. The total sample included 10 participants with an average age of 48.7 (SD 18.6) years. The sample comprised largely female (8/10, 80%), Black (8/10, 80%), and non-Hispanic or Latino (9/10, 90%) participants, and had no children living at home (9/10, 90%). Most reported completing college (6/10, 60%) and either full-time or part-time employment at the time of usability testing. Half of the sample (5/10, 50%) reported <US $50,000 annual household income, and only 30% (3/10) of the participants reported never being married.

**Table 1:** Demographic characteristics of the usability testing participants (N=10).

Demographics			Values
Age (years), mean (SD)			48.7 (18.6)
**Sex, n (%)**			
	Female	8 (80)	
**Race, n (%)**			
	Black or African American	8 (80)	
	Non-Hispanic or Latino	1 (10)	
	Other	1 (10)	
**Education, n (%)**			
	Some college	4 (40)	
	College graduate	3 (30)	
	Postgraduate work	3 (30)	
**Household annual income (US $), n (%)**			
	<50,000	5 (50)	
	≥50,000	5 (50)	
**Marital status, n (%)**			
	Single (never married)	3 (30)	
	Married	4 (40)	
	Divorced	3 (30)	
**Children living at home, n (%)**			
	None	9 (90)	
	≥1	1 (10)	

### Quantitative Results

The usability testing survey that was conducted after 2 weeks of IVR system use yielded positive results (Table 2). All participants (10/10, 100%) agreed that the IVR system was easy to use without the need for technical assistance or extensive learning, and most (7/10, 70%) would recommend the IVR system to others. The participants were confident in using IVR (8/10, 80%), and 70% (7/10) would like to use IVR frequently. Very few participants found the IVR system cumbersome (2/10, 20%) or confusing (3/10, 30%), and only 10% (1/10) of the participants found the IVR system to be unnecessarily complex. In terms of functionality, 70% (7/10) of the participants agreed that the various functions of the IVR system were well-integrated. The participants (7/10, 70%) reported receiving their calls at the scheduled time, and 90% (9/10) reported a female voice on their calls. Only 40% (4/10) of the participants reported wearing the study pedometer; however, of the 60% (6/10) who did not wear the study pedometer, 50% (3/6) used an Apple Watch and 50% (3/6) used a Fitbit Inspire. To numerically interpret the usability of the system, the standardized System Usability Scale scoring procedure was used [19]. This resulted in an average score of 81 (SD 5). Previous research indicates that a System Usability Scale score of >68 can be considered as above-average usability. This score of 81 translates to an e*xcellent* usability rating [20].

**Table 2:** Usability testing survey results (N=10).

Statement and answers^a^				Participants, n (%)
**I think I would like to use this phone counseling system frequently.**				
	Somewhat agree			7 (70)
	Neither agree nor disagree			3 (30)
**I found the phone counseling system unnecessarily complex.**				
	Strongly disagree			7 (70)
	Somewhat disagree			2 (20)
	Somewhat agree			1 (10)
**I thought the phone counseling system was easy to use.**				
	Strongly agree			5 (50)
	Somewhat agree			5 (50)
**I think that I would need the support of a technical person to be able to use this system.**				
	Strongly disagree			7 (70)
	Somewhat disagree			3 (30)
**I found the various functions in this phone counseling system were well-integrated.**				
	Strongly agree			3 (30)
	Somewhat agree			4 (40)
	Somewhat disagree			3 (30)
**I thought there was too much inconsistency in this phone counseling system.**				
	Strongly disagree			4 (40)
	Somewhat disagree			3 (30)
	Neither agree nor disagree			2 (20)
	Somewhat agree			1 (10)
**I would imagine that most people would learn to use this phone counseling system very quickly.**				
	Strongly agree			4 (40)
	Somewhat agree			6 (60)
**I found the phone counseling system very cumbersome to use.**				
	Strongly disagree			4 (40)
	Somewhat disagree			4 (40)
	Neither agree nor disagree			1 (10)
	Somewhat agree			1 (10)
**I felt very confident using the phone counseling system.**				
	Strongly agree			6 (60)
	Somewhat agree			2 (20)
	Neither agree nor disagree			1 (10)
	Somewhat disagree			1 (10)
**I needed to learn a lot of things before I could get going with this phone counseling system.**				
	Strongly disagree			5 (50)
	Somewhat disagree			5 (50)
**How likely are you to recommend this system to others? (Scale of 0-10)**				
	2			2 (20)
	6			1 (10)
	8			4 (40)
	9			2 (20)
	10			1 (10)
**Did you receive your calls at the scheduled time?**				
	Yes			7 (70)
	No			3 (30)
**What gender was the voice on your calls?**				
	Female			9 (90)
	Both male and female			1 (10)
**Did you use the study-provided pedometer to track your steps?**				
	Yes			4 (40)
	**No**			6 (60)
		Apple Watch	3 (30)	
		Fitbit Inspire	3 (30)	

^a^Please select the answer that best expresses how you feel about each statement after using the phone counseling system over the past 2 weeks.

### Qualitative Results

A total of 5 overarching themes emerged: (1) likes or dislikes of the intervention, (2) barriers to or facilitators of PA, (3) technical difficulties, (4) quality of the calls, and (5) suggestions for improvement of the intervention.

#### Likes and Dislikes About the IVR Intervention

When asked what they liked about the DIAL intervention, several participants stated that the phone calls motivated them to exercise and kept them accountable:

I wasn't as active, but after I went through the calls, I became more active and aware, and I was becoming used to the calls, and I was looking forward to the calls, and I was looking forward to the motivational tips.

I think because it held me accountable. The accountability to hear what I had accomplished and what I not accomplished, that adds extra value because it almost puts a mirror in front of your face, and says, “Look.” Sometimes it's very difficult to look at that mirror, and say, “This is what I have or have not done.”

The participants also appreciated the flexibility of the (new) bidirectional call format:

One thing I did like was that, for instance, if I did not make my call. I had the opportunity to call back. That was good.

You have the different options and different times of calling, I think that's good for the people that's busy. So if they miss the call, they can call back, or the system will call them back, but if they need to change their time of the call, then they able to do that.

Finally, the participants looked forward to the PA tips at the end of the call:

I think motivating tips at the end, they were good. I knew them already, but I listened to them. So I think that they were good for people that's just starting out with their health journey.

Regarding dislikes, the participants expressed concerns that specific step feedback messages were *negative* and *stern.* For example, when <10,000 steps per day were reported in the PA-tracking call, the participants received the following feedback:

Thanks for reporting your steps. You did not meet the DIAL study step goal of 10,000 steps per day yet, but you are on your way. Keep making small increases until you get there.

The participants had strong reactions to this feedback and compared it to *a slap in the face*:

It would be a little discouraging to hear that every day, “You didn't meet your 10,000 steps goal. You did not meet the goal. You did not meet the goal.”

The incentives for IVR call completion were another dislike, particularly for rural county coordinators:

I don't think that that 25 cents is helpful for motivating people to continue to get the call.

What’s with the incentive? That's kind of really, make you feel a little worthless.

Other participants were more open to the idea:

Anything that's an incentive that would give people the extra motivation to want to do it, I think it's a good idea...it's not much, but it gives you that sense of, “I made it. I got a quarter, I got 50 cents.” It's not much, but it's that knowing that something is in [it at] the end for you.

#### Facilitators of and Barriers to Participating in PA or the IVR Intervention

Chronic disease prevention and management was an important motivator for participation in PA or the IVR intervention:

I think depending on where people are in their lives, being physically active might be motivated by so saying, “Hey, this disease process can be kept at bay or managed or maybe even prevented if you exercise.”

Social support was also key to encouraging PA initiation and maintenance, especially once the DIAL intervention ended:

Yes, I do think that they will start or to continue to exercise if they have a friend or a buddy to walk with or whatever. I think that that is important to have someone to exercise with.

I think that the interpersonal aspect of it will be really important. Although it's not a person, when that connection and accountability with the phone system is removed, I think it will be really important to have that from another source, and hopefully other participants or family members of the participants.

As for barriers to engaging in PA and completing the IVR calls, the participants stressed the lack of time and competing interests:

There are many, many days where I don't want to do any physical activity. I would say actually most days. It's not because it's tedious. It's because I have so many things to do, and I keep thinking, “Wow. I got to spend that hour doing this.”

#### Technical Difficulties

The participants described experiencing some initial technical difficulties with the IVR calls, such as receiving calls at incorrect times or with *system error* messages. The programming decision to skip calls on holidays also seemed to cause some confusion and was changed as a result:

During the 4th of July holidays, I didn't receive any calls at all that weekend.

Finally, the participants learned to take their time entering the responses during the IVR calls:

If you trying to speed it up and hurry up, you know you going to press two, nuh-uh. it's going to hit you with an error.

#### Quality of the Calls

The participants generally indicated that the *quality of the call is good*. In fact, rural county coordinators had previously given the Amazon Polly narration a favorable review at a focus group. During usability testing, several participants distinguished the female voice options as *less monotone, robotish* than certain *boring* male voice options. The pace of the calls received mixed reviews; *It really was a good pace* for some and *a bit too fast* for others:

There were times where it felt like it was moving a bit too fast, especially when there were multiple options or the question or the prompt was read or said, and then the answers were said immediately after. I don't know, sometimes it was rushed through, it felt like.

#### Suggestions

The participants stated that they preferred to have written *user-friendly* instruction materials that could be used during the calls:

I think you can give them a little prompt card. Like some of the prompts. Because it's just different every time, but just a small, little introduction of what to expect.

The participants also suggested that having printed materials of the survey readily available to community participants would be beneficial and crucial for capturing accurate data:

I think it would help to have some sort of printout. Just a scale that says, “This is what one means, and this is what 100 means.” Because again, and maybe I just was doing too many things sometimes. For me to remember what that scale was. If I had a call that was coming through, of course I would not click over, but that's a distraction for me, again. So if I'm in the middle of a question, I'm like, “Oh my gosh. Am I supposed to pick 1 or 100.” I think a printout scale or something in front of me probably would have reminded me, because again, some days I'm putting one, some days I'm putting 99, and that's not what I meant. But there was no way for me to go back and erase my answer, to my knowledge. I don't know if I missed that in the training, but I just thought, “Oops.” But that was just my short term memory, and knowing I needed to complete the call.

In addition to the advantage of obtaining accurate data, the participants stated that having printed materials would also mitigate noncompliance from frustration:

Anything that we could get to assist would always be helpful. So, if we could come up with something, some type of visual aid for the older generation, then that will be great. I'm sure everybody can work a phone, but you want to make sure that they're not getting confused. Because once they get confused, confusion causes discouraged sometimes. So, you don't want to get them confused. So, yeah, if we could come up with some type of handout that would be great.

The participants suggested that step goals should be modest and community participants should be reminded to gradually increase their steps during the study:

Take baby steps. I think if you want to see somebody make it to that 10,000 goal marker, try to start small, like see where they're at and see what is an average for the participant and then work your way up from there. And ultimately, it may be just too hard for somebody's daily schedule to meet that 10,000 goal step without making significant changes to their daily routines. But I think if you take baby steps, then for a good bit of the participants, you may not got to do it for everybody, but at least a certain group of the participants, and you're able to get some changes, like an increase in daily steps from them, they're all meeting the goals, then I think that could be a good motivating factor.

It did tell me, try to add a 500 steps for the next time, but I feel like it should be more personalized like, “Okay, you got 2000, tomorrow let's try to get 2,500.” And then when they call the next day, if they have the 2,500 be like, “Great, you met the goal. Do you think you could add another 500?” I feel like that's how it should be. I don't feel like the bar should right off the top be 10,000 because that's a lot for some people.

The participants also provided solutions for the previously mentioned issues with incentives for call completion (eg, substituting nonmonetary incentives and a point system):

I can tell you that getting 25 cents for each phone call was not motivating at all. Yeah. I don't mean to be blunt, but it wasn't. It wasn't motivating. How much I earned at the end of each phone call, it just didn't motivate me. Now, if I earned points for each phone call, and I could redeem those points in some sort of physical activity, online store.

I mean, the value may be still $3.75, but with let's just say 375 points may be like a gift. It may be a pedometer. It may be a little lunch tote, or it may be something else. People can use a tote. They can use a pedometer. They can use even a cup or a mug, or a water bottle if they're exercising. But what can you do with $3.75?

Finally, the participants discussed the tips provided to increase the number of steps and suggested that they be personalized to the Deep South rural community:

For instance, tell them like get up during commercial break and walk around your coffee table twice. That's feasible. It's within reach and it doesn't take a lot of effort to go out. Because think about these people that don't have parks nearby. We're telling them to go to the nearest park. Well, there is no nearest park.

Tell them, okay, well walk around your house two times or walk to the mailbox twice or for instance what's something else. Go three mailboxes down and come back. Something that people can be like, “Oh yeah, I can do that. I never thought to do that.”

## Discussion

### Summary

Innovative IVR systems hold the potential to overcome barriers to achieving the recommended levels of PA in the rural Black Belt region of Alabama [3]. However, no previous research has examined IVR systems in rural contexts to increase PA levels. We developed an IVR system in an iterative manner based on feedback from earlier pilot studies, focus groups, and the current usability testing with key stakeholders (both community members and local county coordinators with UAB O’Neal Comprehensive Cancer Center Community Outreach and Engagement Office). The resultant system was characterized by high usability and is currently being tested for efficacy in an RCT.

### Principal Findings and Resultant IVR System

The IVR system received a numerical usability score of 81—equating to an e*xcellent* usability score. The sequential explanatory mixed methods design we adopted helped us identify several opportunities for improvement through the qualitative interviews. After usability testing (qualitative interviews), we implemented several improvements into the system. First, we modified our messaging when the participants failed to reach their goals to sound less negative or stern. We implemented graceful handling of wrong key presses by participants; instead of informing them that they had pressed a wrong key, we reworded to say that the system could not understand. We implemented a detailed orientation session procedure in which the IVR system was oriented and printed materials were made available. Instead of directly pushing the participants toward 10,000 steps, following participant feedback, we implemented incremental goals of 250 steps per week. We reworded our reward system to use the word *points* instead of *cents* to emphasize the gamification of IVR adherence versus financial transactions. Finally, we also added several more PA tips as suggested by our interview participants.

As a means to further test the system before the commencement of the RCT, the core group of researchers working on this study met to discuss whether further formal usability testing was required. As most reported issues pertained to wording or content, it was decided that no further usability testing was needed. However, the core group of researchers were listed as pilot users of the IVR system. These pilot users were scheduled approximately 2-3 weeks ahead of the actual RCT participants. These researchers actively tested the system daily and reported to the development team on any issue found. This enabled the development team to aggressively address the issues before any RCT study participants encountered them. Some example issues identified and fixed using this approach included problems when the participants moved from one phase (daily calls) to another (biweekly calls) and nonavailability of new PA strategies to suggest to participants. Our *2 weeks ahead approach* enabled us to resolve these issues before any real participants encountered them while avoiding lapses in time that would delay the project.

Our final product is a comprehensive IVR system with cutting-edge capabilities such as streamlined calls, smart dropped calls handling, and assignable voice gender. Future research should examine the added value of such features and their impact on this promising technology.

### Final IVR System Design

The feedback during, before, and after the usability testing was used to iteratively refine the IVR system. The resultant system, which is now being used in the RCT, is described as follows:

1. The system is designed to handle complex call schedules involving different types of calls during different phases of the intervention and the randomization group.
2. The system can receive incoming calls and smartly place outgoing calls only if the participant has a pending incomplete call.
3. To protect the privacy of the participants, they are identified using their phone number and a personal identification number (PIN). When the participants use their registered phone, only a PIN is required. When the participants use a phone other than their registered phone, both the registered phone number and PIN are required. This achieves a balance between user experience and security.
4. New participants are registered on a web portal by a study manager who retrieves the unique PIN for the user. The study manager is then able to print the PIN and other instructional materials in an educational binder for the participants.
5. A comprehensive missed call policy has been implemented, with the system retrying the call after 30 minutes. Again, if there is no response, the call is marked as incomplete and can be completed the next day.
6. A smart dropped-call policy has also been implemented, wherein if a participant drops midway through a call and the user connects again within a preset time limit, the participant is able to continue from the last question they answered.
7. One of the most significant aspects affecting the usability of IVR systems is the information navigation time [7]. We have essentially eliminated the navigation time by streaming the content for calls in multiple ways. First, calls are not placed unless there is a pending survey to be completed. Second, when users have multiple surveys due, the system combines all the surveys and offers them in a sequence. Finally, if the participant has any pending surveys as a result of missed calls in the previous 2 days, the system offers the missed surveys in sequence.
8. Many IVR systems require a significant amount of time because of the confirmation messages, such as “You pressed 6, press 1 if this is right or press 2 to change.” These confirmation messages are necessary as it is easy for a participant to accidentally mistype a number; however, these confirmation messages almost double the call time. To overcome this, during the orientation session, we educate the users on pressing * anytime during the call to edit the last response.
9. The participants can call the IVR system anytime and change their preferred call receiving time.
10. To maximize information relevance [7], the system is programmed to be able to look up the participant’s previous step goals, PA self-efficacy, enjoyment, social support, and outcome expectancies and use those values as a part of the conversation—thereby leading to high relevance.
11. To maximize information capacity [7], a bank of PA-increasing strategies has been created, with new strategies being revealed on a weekly basis. Similarly, a bank of greeting messages has also been made available. Through these mechanisms, despite the daily calls during the first 3 months, the users would find a variety of content being delivered.
12. At the end of each call, the system announces the reward points earned by the participant, which can be redeemed for actual monetary incentives. Before the usability testing, we directly referred to points as cents. However, we learned from usability testing that the participants felt that 25 cents per call made them feel that their time was worthless. Thus, we reworded our call content to award points rather than cents.
13. The gender of the voice narration in the call can be set to match the gender of the participant, to the opposite gender, or to be random.

### Strengths and Limitations

This study had a few limitations. First, usability testing was conducted during the COVID-19 pandemic; thus, for participant safety, all surveys and interviews were conducted remotely, and participation was limited to community health advisors and staff. Although these community health advisors live and work in the same rural counties of the Black Belt region of Alabama as the future participants, it is possible that they do not accurately represent the demographics of the participants (eg, education levels) who would participate in the RCT study. In addition, our demographics includes predominantly female and non-Hispanic or Latino populations.

However, this opportunity allowed rural county coordinators to gain familiarity and comfort with the inner workings of the IVR system before spearheading its dissemination among their own communities. Thus, they will be more prepared to orient participants to the IVR system and field their questions. Moreover, playing such a key role in the development and refinement of this technology likely enhanced the sense of buy-in and ownership among these key stakeholders and gatekeepers to the community and substantially improved the final product.

### Conclusions

This study demonstrated that the developed IVR system is usable and has the potential to increase the levels of PA. Study findings provided insight into the participants’ preferred language, narration tones, rewards, and variety of messaging. These insights can be valuable for future studies that seek to develop IVR-based interventions.

## Abbreviations

DIAL: Deep South IVR-Supported Active Lifestyle
IVR: interactive voice response
PA: physical activity
PIN: personal identification number
RCT: randomized controlled trial
SCT: social cognitive theory
UAB: University of Alabama at Birmingham

## References

[1] Gyawu R, Quansah JE, Fall S, Gichuhi PN, Bovell-Benjamin AC (2015). Community food environment measures in the Alabama Black Belt: implications for cancer risk reduction. Prev Med Rep.

[2] Pekmezi D, Marcus B, Meneses K, Baskin ML, Ard JD, Martin MY, Adams N, Robinson C, Demark-Wahnefried W (2013). Developing an intervention to address physical activity barriers for African-American women in the deep south (USA). Womens Health (Lond).

[3] Tsoli S, Sutton S, Kassavou A (2018). Interactive voice response interventions targeting behaviour change: a systematic literature review with meta-analysis and meta-regression. BMJ Open.

[4] Wireless Substitution: Early Release of Estimates from the National Health Interview Survey, January – June 2021. National Center for Health Statistics.

[5] Kassavou A, Sutton S (2018). Automated telecommunication interventions to promote adherence to cardio-metabolic medications: meta-analysis of effectiveness and meta-regression of behaviour change techniques. Health Psychol Rev.

[6] Posadzki P, Mastellos N, Ryan R, Gunn L, Felix L, Pappas Y, Gagnon M-P, Julious SA, Xiang L, Oldenburg B, Car J (2016). Automated telephone communication systems for preventive healthcare and management of long-term conditions. Cochrane Database Syst Rev.

[7] Lewis J (2006). Usability testing. Handbook of Human Factors and Ergonomics, Third Edition.

[8] Asthana S, Singh P, Singh A (2013). Exploring the usability of interactive voice response system's design. Proceedings of the 3rd ACM Symposium on Computing for Development.

[9] Cilliers L, Flowerday S (2017). Factors that influence the usability of a participatory IVR crowdsourcing system in a smart city. South African Comput J.

[10] Lerer A, Ward M, Amarasinghe S (2010). Evaluation of IVR data collection UIs for untrained rural users. Proceedings of the First ACM Symposium on Computing for Development.

[11] Brown NI, Powell MA, Baskin M, Oster R, Demark-Wahnefried W, Hardy C, Pisu M, Thirumalai M, Townsend S, Neal WN, Rogers LQ, Pekmezi D (2021). Design and rationale for the deep south interactive voice response system-supported active lifestyle study: protocol for a randomized controlled trial. JMIR Res Protoc.

[12] Bandura A (1989). Human agency in social cognitive theory. Am Psychol.

[13] Ainsworth M (2020). Effects of Interactive Voice Response (IVR) counseling on physical activity benefits and barriers. Health Behav Policy Rev.

[14] Pekmezi D, Ainsworth C, Holly T, Williams V, Joseph R, Wang K, Rogers LQ, Marcus B, Desmond R, Demark-Wahnefried W (2018). Physical activity and related psychosocial outcomes from a pilot randomized trial of an interactive voice response system-supported intervention in the deep south. Health Educ Behav.

[15] Pekmezi D, Ainsworth C, Holly T, Williams V, Benitez T, Wang K, Rogers LQ, Marcus B, Demark-Wahnefried W (2017). Rationale, design, and baseline findings from a pilot randomized trial of an IVR-Supported physical activity intervention for cancer prevention in the Deep South: the DIAL study. Contemp Clin Trials Commun.

[16] Braun V, Clarke V (2006). Using thematic analysis in psychology. Qual Res Psychol.

[17] Zamawe F (2015). The implication of using NVivo software in qualitative data analysis: evidence-based reflections. Malawi Med J.

[18] Thurmond VA (2001). The point of triangulation. J Nurs Scholarsh.

[19] Brooke J (1996). SUS: a "quick and dirty" usability scale. Usability Evaluation In Industry.

[20] Bangor A, Kortum P, Miller J (2009). Determining what individual SUS scores mean: adding an adjective rating scale. J Usability Stud.

